# A Practical Genome Scan for Population-Specific Strong Selective Sweeps That Have Reached Fixation

**DOI:** 10.1371/journal.pone.0000286

**Published:** 2007-03-14

**Authors:** Ryosuke Kimura, Akihiro Fujimoto, Katsushi Tokunaga, Jun Ohashi

**Affiliations:** 1 Department of Human Genetics, Graduate School of Medicine, The University of Tokyo, Tokyo, Japan; 2 Japan Society for the Promotion of Science, Tokyo, Japan; University of Utah, United States of America

## Abstract

Phenotypic divergences between modern human populations have developed as a result of genetic adaptation to local environments over the past 100,000 years. To identify genes involved in population-specific phenotypes, it is necessary to detect signatures of recent positive selection in the human genome. Although detection of elongated linkage disequilibrium (LD) has been a powerful tool in the field of evolutionary genetics, current LD-based approaches are not applicable to already fixed loci. Here, we report a method of scanning for population-specific strong selective sweeps that have reached fixation. In this method, genome-wide SNP data is used to analyze differences in the haplotype frequency, nucleotide diversity, and LD between populations, using the ratio of haplotype homozygosity between populations. To estimate the detection power of the statistics used in this study, we performed computer simulations and found that these tests are relatively robust against the density of typed SNPs and demographic parameters if the advantageous allele has reached fixation. Therefore, we could determine the threshold for maintaining high detection power, regardless of SNP density and demographic history. When this method was applied to the HapMap data, it was able to identify the candidates of population-specific strong selective sweeps more efficiently than the outlier approach that depends on the empirical distribution. This study, confirming strong positive selection on genes previously reported to be associated with specific phenotypes, also identifies other candidates that are likely to contribute to phenotypic differences between human populations.

## Introduction

Modern human populations exhibit large phenotypic differences in morphological and physiological traits such as pigmentation, hair shape, body shape and composition, and enzymatic activities. Such phenotypic divergences between populations are considered to result from genetic adaptation to local environments; people have experienced changes in their environment due to migration and establishment of civilizations over the past 100,000 years. In order to reveal the genetic bases of population-specific phenotypes, it is necessary to decode the signatures of gene histories engraved on the human genome. In one important “engraving” process known as the “hitchhiking effect”, positive selection of an advantageous mutation alters patterns of genetic variation in the adjacent sequence [1]. During fixation of an advantageous mutation, this process causes a “selective sweep,” i.e., elimination of variation in the neighboring region. To detect recent hitchhiking events, one practical approach is to search for genetic regions that have undergone few previous recombination events [2]. Another selection clue is high local genetic differentiation that can be measured with F_ST_ [3] when at least two populations are examined. To detect loci that have reached fixation, low nucleotide diversity should be considered along with the aforementioned points.

To date, numerous different approaches have been applied to genome-wide scans for positively selected loci in humans. Some of these rely on the McDonald-Kreitman test [4] or the dn/ds test [5], in which nucleotide sequences are compared with those of the chimpanzee [6]–[8]. Other techniques employ polymorphisms within species, and mainly utilize summary statistics such as F_ST_, heterozygosity, or neutrality tests based on the site frequency spectrum (Tajima's D [9], Fu and Li's D [10], Fay and Wu's H [11], and Kim and Stephan's composite likelihood tests [12]), as well as linkage disequilibrium (LD)-based statistics [13]–[23]. In particular, LD-based methods using the concept of extended haplotype homozygosity (EHH) have been frequently employed because of their power for detecting a rapid increase in the frequency of an advantageous mutation under recent selection [24], [25]. However, since these approaches require at least two alleles for comparison within a population, they are not applicable to already fixed loci, which are most likely to be under strong positive selection. In addition, when applied to a genome-wide scan for candidate loci, the current methods must be applied to every polymorphism or core region that may exhibit strong LD with another.

Since summary statistics are usually affected by population demographic history, in previous genome-wide analyses, candidates of selected loci were identified at the extreme tails of empirical distributions. However, recent studies have suggested that such outlier approaches provide a number of false positive loci because of the large size of the human genome [26], [27]. Given the trade-off that exists between Type I error rate and the power to detect true selected loci, the appearance of false positives is inevitable. Even so, improving current detection methods would certainly reduce the number of false positives. For example, one reason for the loss of detection power and the generation of false positives is the heterogeneity of recombination rates across the human genome [28]. In some previous methods, the genome is divided into windows of constant physical size in order to calculate summary statistics. Such methods can be complicated by the heterogeneity of recombination rates. Therefore, local recombination rates should be considered when window sizes are defined.

In order to reduce the limitations of the previous LD-based approaches, we developed a method for scanning genome-wide SNP genotype data for population-specific selective sweeps that have reached fixation, in which homozygosity for haplotypes in two populations are compared. In addition, to measure haplotype differentiation between populations, we compared two populations in homozygosity for the most frequent haplotype instead of using F_ST_. Although SNP genotype data do not always include selected polymorphisms, our method can efficiently detect the region where a selected polymorphism is located. Another advantage of our method is that the use of observed values of homozygosities allows us to omit haplotype phasing that needs to be either determined from family data or estimated from population data by means of a time-consuming computation.

## Results

### The ratio of haplotype homozygosity between populations

**Figure 1:**
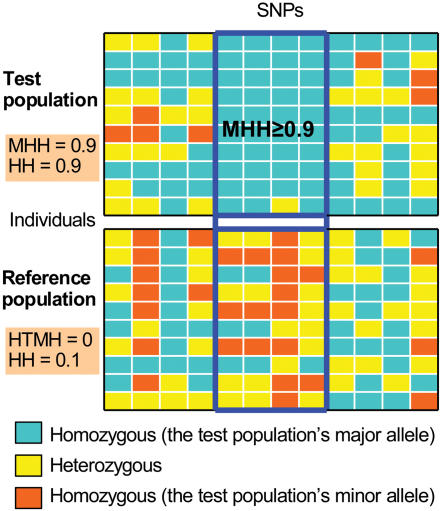
Block partition based on MHH. Blocks were defined as containing at least two SNPs with the most frequent haplotype homozygosity (MHH) ≥0.9 in the test population (blue frame). Using these same blocks, homozygosity for the test population's most frequent haplotype (HTMH) in the reference population, and haplotype homozygosity (HH) were calculated in both the reference and test populations. This illustration shows the observed values of haplotype homozygosities, *i.e*., the frequencies of homozygous diplotype individuals in each population. In the test population, 9/10 individuals have homozygous genotype for all the SNPs in the block, *i.e.*, four blue boxes. In the reference population, only 1/10 individual have homozygous diplotype (two blue and two red boxes) and no individual is homozygote for the test population's most frequent haplotype (four blue boxes).

To identify population-specific selective sweeps, we used data from multiple populations, where one population is selected as the test population and another is used as the reference population (Fig. 1). Statistics used in our method are haplotype homozygosity (HH), the probability that two haplotypes sampled at random from the population are same, and most frequent haplotype homozygosity (MHH), the probability that both the two haplotypes sampled are the most frequent haplotype in the population. Here, two sequences that have the same nucleotide for all the typed SNPs were regarded as the same haplotype. HH and MHH can be estimated as observed values (ΣX_ii_ and X_11_) or expected values (Σx_i_^2^ and x_1_^2^), where X_ii_ and x_i_ denote the frequency of individuals homozygous for the i^th^ frequent haplotype and the frequency of the i^th^ frequent haplotype, respectively. When we use the observed values, we do not have to determine or estimate haplotype phase.

The first process of our method is to define blocks for calculation of the statistics by the use of MHH (or HH) itself (Fig. 1). The block partition is made using the data of the test population in order to capture regions with low haplotype diversity. Because we are interested in population-specific fixations in this study, we set the definition of blocks as having at least two SNPs with MHH ≥0.9, which corresponds to the frequency of the most frequent haplotype ≥ approximately 0.95. Blocks were partitioned in the direction from small to large number of the chromosomal nucleotide position. Then, using the same block, we calculate homozygosity for the test population's most frequent haplotype (HTMH) in the reference population, and HH in both the reference and test populations.

To detect population-specific change in haplotype frequency, the ratio of MHH (rMHH) is calculated as
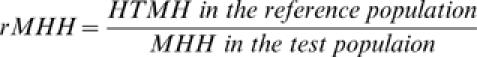
1If the test population's most frequent haplotype is rare in the reference population, rMHH shows an extremely small value, which may occur when the frequency of an advantageous mutation increased only in the test population. Therefore, this value is inversely correlated with the extent of local haplotype differentiation between populations. On the other hand, the ratio of HH (rHH) is calculated as
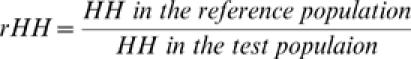
2If a region showing MHH ≥0.9 is unusually extended in the test population, rHH exhibits a small value. Therefore, rHH represents the extent of the population-specific decrease in haplotype diversity and thus can be an index for detecting a recent rapid increase in the frequency of an allele. Here, we can control the heterogeneity of recombination rates if the reference population can be regarded as neutral. However, rMHH and rHH would not show small values in the loci where the same allele is selected and fixed in both populations used in the comparison. In case different mutations in the same region were selected in the two populations, rMHH would show a small value, but rHH would not.

### Power and false positives in detection of selection with rMHH and rHH

**Figure 2:**
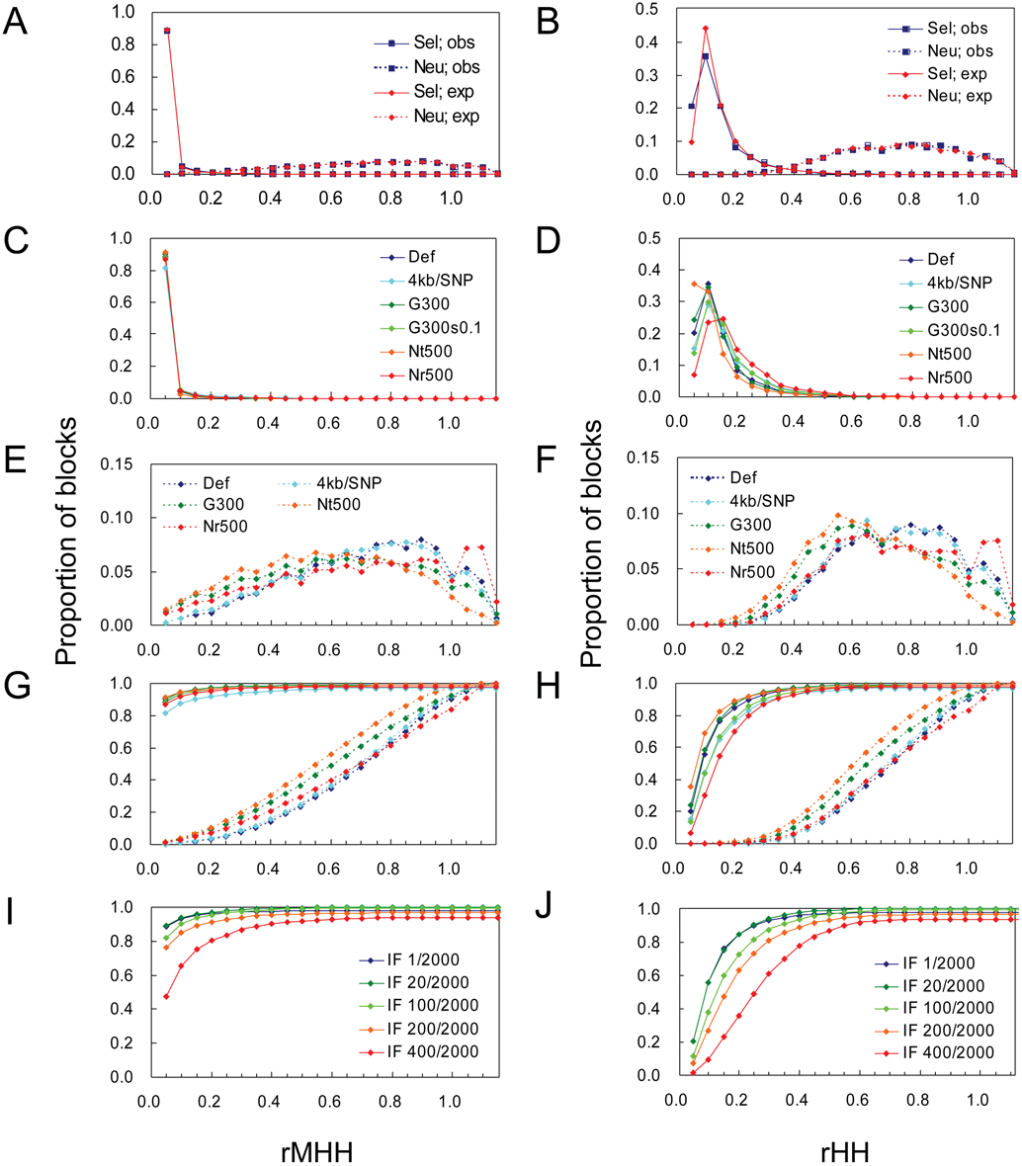
Distributions of rMHH and rHH under simulations. (A and B) Comparison of the observed (obs) and expected (exp) values of homozygosities. Distributions of rMHH (A) and rHH (B) under the selection (sel) and neutral (neu) models were shown. The parameters used in the simulations are default settings (def): size of the test population, N_t_ = 1000; size of the reference population, N_r_ = 1000; generations after the population split, G = 200; selection coefficient, s = 0.15; SNP interval = 2 kb/SNP. (C and D) rMHH (C) and rHH (D) under different selection models. (E and F) rMHH (E) and rHH (F) under neutral different models. The parameters were changed accordingly in these simulation models. (G and H) Accumulative distributions of rMHH (G) and rHH (H). Denotation of each line is same as in C–F. (I and J) Accumulative distributions of rMHH (I) and rHH (J) for the case of selection on a standing allele. Selection models for several initial frequencies (IF) of the advantageous allele and a neutral model (neu) were analyzed.

To estimate the detection power of rMHH and rHH, we performed computer simulations (Fig. S1). In the simulations, we assumed two divergent populations: one is the reference population under neutral conditions, and the other is the test population either under neutral conditions for null distribution or under genic selection on the derived allele. In the selection model, we assumed strong selective pressure so that the advantageous allele reaches fixation. The selected polymorphism was not used for calculation of the statistics. We first compared the observed and expected values of homozygosities. When we considered the case that a new advantageous mutation has fixed, rMHH and rHH exhibited low values regardless of the way of homozygosity estimation as shown in Fig. 2A and B, respectively. The observed and expected values showed the same distribution when the test population is neutral as well. Thus, we used the observed values of MHH and HH in the analyses described below. When we evaluated the effect of the density of typed SNPs, we found neither statistic was much affected (Fig 2C and D). In addition, under strong selective sweeps resulting in fixation, the distribution of rMHH was robust against changes in strength of selection and demographic parameters such as generations after divergence and population size (Fig 2C), whereas the distribution of rHH was susceptible to the difference in population size between the test and reference populations (Fig 2D). In the case of the test population under neutral condition, demographic parameters had a great influence on the distribution of both rMHH and rHH (Fig. 2E and F). This indicates that an increased number of generations after divergence and a decreased size of the test population raise the number of false positives due to genetic drift when we set certain threshold values of rMHH and rHH. In all the cases simulated, rMHH<0.05 and rHH<0.3 corresponded to approximately 90% detection power (81.5%–91.5% for rMHH and 86.8%–94.5% for rHH) (Fig 2G and H). Therefore, we used these values as thresholds in subsequent analyses. Type I error rates for rMHH<0.05 and rHH<0.3 ranged from 0.24%–1.43% and 0.76%–4.62%, respectively.

**Figure 3:**
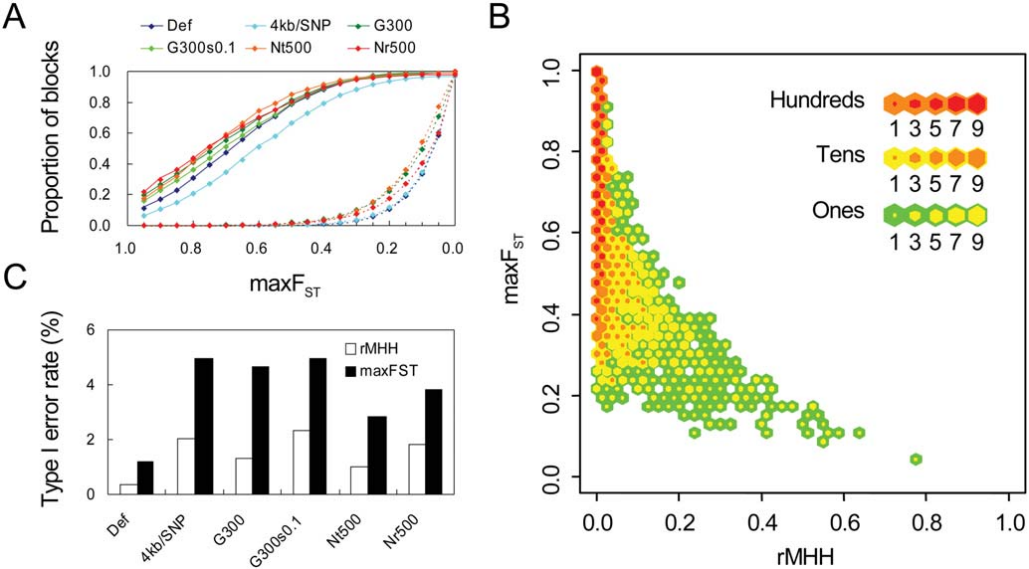
Comparison between rMHH and maxF_ST_. (A) Accumulative distribution of maxF_ST_. (B) The distribution of rMHH and maxF_ST_ under a selection model (default settings as in Fig. 2). (C) Type I error rate of rMHH and maxF_ST_ at the threshold of 90% detection power under different models. The difference between rMHH and maxF_ST_ was significant (P<10^−17^ to 10^−100^).

To examine the ability of rMHH to capture genetic differentiation without typing of the selected polymorphism, we compared rMHH with maxF_ST_, *i.e*., the highest F_ST_ for all the typed SNPs in the block. In contrast to rMHH, maxF_ST_ was strongly affected by the density of typed SNPs when the test population was under selection models (Fig 3A). Fig. 3B indicates that rMHH is inversely correlated with maxF_ST_, showing an ability to capture highly differentiated regions where the maxF_ST_ is low. When exactly 90% detection power was assured for both the statistics, maxF_ST_ yielded false positives twice more than rMHH for any condition tested (Fig. 3C).

We also considered the case in which natural selection acts on an old standing allele. Here we assumed that a previously neutral derived allele became advantageous after the split of two populations. The distribution of rMHH and rHH depended on the initial frequency of the advantageous allele at the time positive selection began to act. As shown in Fig. 2I and J, the power of rMHH<0.05 and rHH<0.3 gradually decreased as the initial frequency increased. For a 1% initial frequency, regions under selection could be detected with these thresholds, comparable to the case of a new advantageous mutation. For a 20% initial frequency, however, the power drastically decreased. When we considered the effect of the initial frequency of the advantageous allele on the length of blocks, we found their inverse correlation (Fig. S2), which indicates that variable block size as defined in this study is essential for detection of selection events under different situations.

### Analyses of the HapMap data and neutral genome simulation

For analyses of real data, we used autosomal SNP genotype data of 180 unrelated individuals from Phase I (release 16c.1) of the International HapMap Project (60 individuals from Yorba in Ibadan, Nigeria, YRI; 60 individuals of northern and western European ancestry from Utah, CEU; 30 Han Chinese individuals from Beijing, CHB, and 30 Japanese individuals from Tokyo, JPT) [18]. Here, we refer to both of the East Asian groups (CHB and JPT) together as EAS. In addition, we performed a coalescent simulation, with non-uniform recombination rates, using the program and best-fitting demographic model of Schaffner *et al*. [29] (Fig. S3A). From this simulation, a data set imitating genome-size chromosomes (2.7 Gbp) under neutrality was obtained. To empirically correct the ascertainment bias in the HapMap data, the probabilities that SNPs were “genotyped” were determined with the minor allele frequency spectra in the HapMap and the simulation (Fig. S3B).

**Figure 4:**
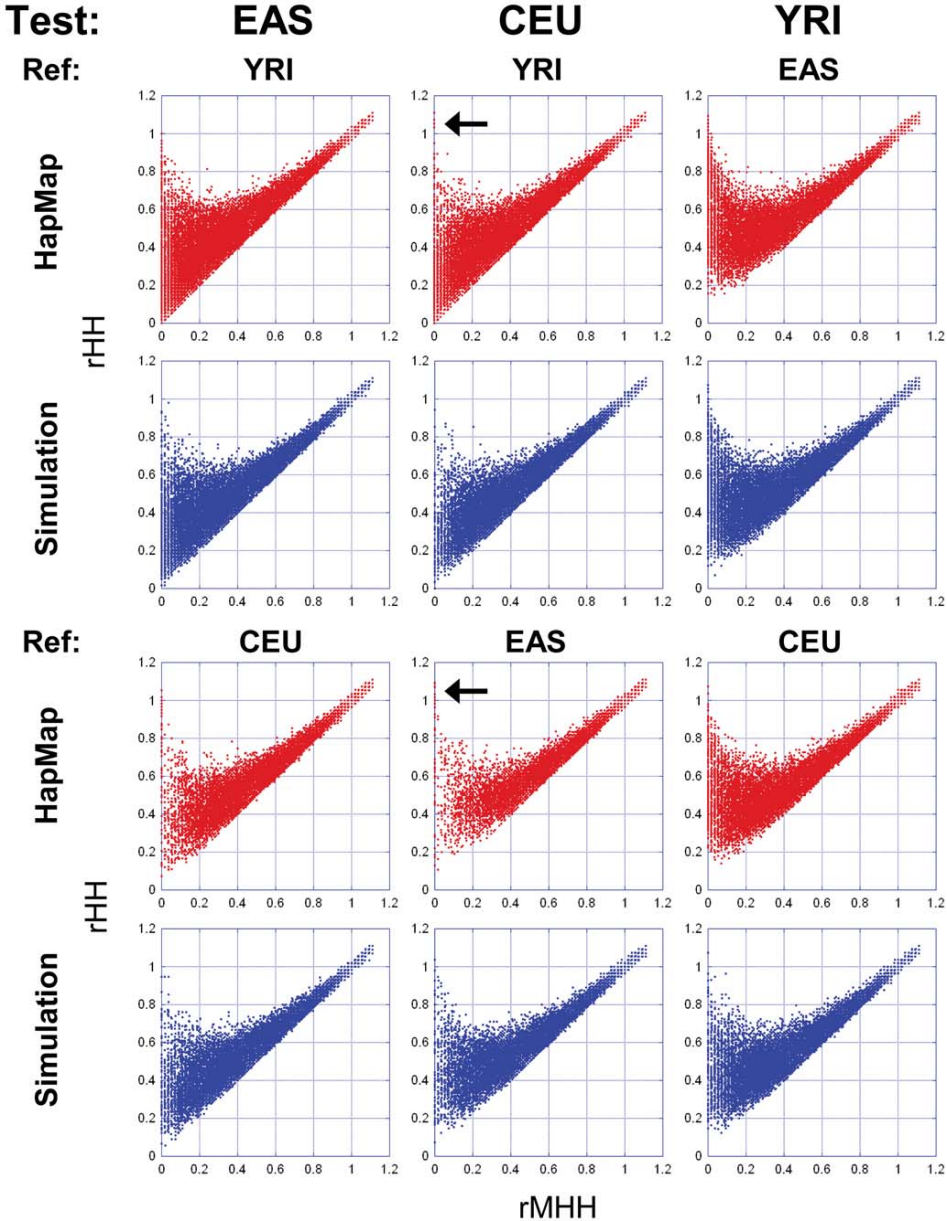
Scatter plots between rMHH and rHH for the HapMap data and neutral genome simulation. Arrows indicates blocks with low rMHH and high HH values, which is rare in the simulation.

We subjected the three populations (YRI, CEU and EAS) to our method in order to detect selective sweeps that have reached fixation or near fixation. Scatter plots between rMHH and physical length of block (Fig. S4) show their inverse correlation but suggest that the elongated block length alone is not adequate for efficient detection of rapid fixations caused by selective sweeps. The number of candidate regions detected is shown in Table 1. Since the distribution of rMHH and rHH in the neutral genome simulation fitted well to that in the HapMap (Fig. 4), this means that the number of detected regions depends on demographic history rather than on natural selection. This result indicates that a relatively large proportion of the candidates are false positives. Many of these false positives probably can be attributed to the distant divergence time between African and non-African populations and to bottleneck events that occurred after the non-African population left Africa. When EAS and CEU were compared with each other as the test and reference populations, a small number of candidates (less than 1% of the defined blocks) were detected (Table 1). By using the combination of rMHH and rHH, we could narrow the candidates down further. Only 12 blocks in EAS, 4 blocks in CEU, and 1 block in YRI satisfied the criterion of rMHH<0.05 and rHH<0.3 for two reference populations (Table 1 and 2). It is worth noting that a few blocks exhibited low rMHH and high rHH in the HapMap CEU data (arrows in Fig. 4), but not in the neutral simulation. Some of these cases are likely to be due to errors in the HapMap project. If they are not due to errors, such blocks might be regions under recurrent fixations; the reference or common ancestor population may also have experienced at least one sweep.

**Table 1:** The number of candidate regions under strong selective sweeps.

Test	Defined blocks	Ref1	Ref1 rMHH<0.05 (A)	Ref1 rHH<0.3 (B)	A∩B	Ref2	Ref2 rMHH<0.05 (C)	Ref2 rHH<0.3 (D)	C∩D	(A∩B)∩(C∩D)
YRI	30237	EAS	843 (2.8%)	210 (0.69%)	31 (0.10%)	CEU	506 (1.7%)	290 (0.96%)	39 (0.13%)	1 (0.0033%)
EAS	53717	YRI	1376 (2.6%)	3505 (6.5%)	874 (1.6%)	CEU	139 (0.26%)	350 (0.65%)	39 (0.073%)	12 (0.022%)
CEU	42709	YRI	657 (1.5%)	1927 (4.5%)	384 (0.90%)	EAS	91 (0.21%)	69 (0.16%)	12 (0.028%)	4 (0.0094%)

Blocks were defined as at least two SNPs with MHH ≥0.9. Candidate regions were detected as blocks with rMHH<0.05 and rHH<0.3.

### Candidates for genes fixed under positive selection

Among regions with strong signatures of selection (Table 2 and Data S1), we observed overlaps with genes that previously had been reported to be targets of strong selection such as *DARC* (*FY*) [30] (YRI (test) vs. CEU (reference)) and *ABCC11* [31] (EAS vs. YRI/CEU) that are associated with malaria resistance and earwax type, respectively. Of four regions with the strongest signatures of European-specific selective sweeps (Table 1), two regions include pigmentation-related genes, *SLC24A5* (*NCKX*) [32] and *SLC45A2* (*MATP*) [33]. When we consulted EntrezGene and OMIM for gene functions, we also found a number of genes that may be involved in certain traits on which natural selection is likely to have acted [34]–[42] (Data S1). For example, *BMP2K* (CEU vs. YRI) is a kinase inducing bone morphogenic protein-2 that participates in skeletal development and patterning. *IGFBP2* (EAS vs. YRI) may have a potential role in growth through IGF-1 action. *EDAR* (EAS vs. YRI/CEU) is related with hair and tooth morphogenesis and *ENAM* (EAS/CEU vs. YRI) is the largest protein in the enamel matrix of developing teeth. Fertility-related genes such as *PGR* (EAS vs. YRI/CEU), *MORC1* (EAS vs. YRI) and *SPAG6* (EAS/CEU vs. YRI) also exhibit strong signatures of selection. *MYLK* (EAS/CEU vs. YRI) and *MYLK2* (EAS vs. YRI) are key enzymes in contraction of smooth and skeletal muscles, respectively. *PET112L* (YRI vs. EAS/CEU) plays an important role in mitochondrial gene expression, most likely in translation. We detected various adiposity-related genes that may be involved in fatty acid metabolism (*PRKAG3*, EAS/CEU vs. YRI), glucose metabolism (*DOK5*, EAS vs. YRI), and vitamin B metabolism (*TPK1*, EAS/CEU vs. YRI). These genes could be candidates for “thrifty genes” that explain variation in the efficiency of energy expenditure among populations [43]. Like pigmentation-related genes, sunlight conditions, which depend on degrees of latitude, may be selective pressures on *PPEF2* (EAS/CEU vs. YRI) involved in photoreception in the visual system and *CSNK1D* (EAS vs. YRI) involved in circadian rhythms and sleep phase. Local epidemic diseases can drive population-specific selection acting on immunity-related genes such as *CD226* (EAS/CEU vs. YRI) and *IL4R* (EAS vs. YRI). It is interesting that two anthrax toxin receptors, *ANTXR1* (CEU vs. YRI) and *ANTXR2* (EAS vs. YRI), show signatures of selective sweeps, which may indicate cases in human history of fights against specific infectious diseases. A drug response-related gene, *CYP3A4* (EAS/CEU vs. YRI), and olfactory receptor genes, *OR3A2* and *OR1G1* (EAS vs. YRI), might also be targets of local natural selection.

**Table 2:** Candidate regions with the strongest signs.

Test	Chr: Position	First_rs..Last_rs	Length	SNP	Ref1	rMHH	rHH	Ref2	rMHH	rHH	Genes
EAS	Chr2:9715614..9721513	rs6432017..rs13426930	5900	5	YRI	0.000	0.232	CEU	0.036	0.214	ADAM17
EAS	Chr2:22298089..22450516	rs396757..rs10196529	152428	51	YRI	0.000	0.000	CEU	0.036	0.125	
EAS	Chr4:36792161..36814135	rs6531489..rs6531493	21975	11	YRI	0.000	0.276	CEU	0.034	0.276	
EAS	Chr5:117480167..117642506	rs7703873..rs1479211	162340	77	YRI	0.000	0.091	CEU	0.000	0.073	
EAS	Chr6:100307973..100327281	rs6916883..rs4640896	19309	7	YRI	0.037	0.148	CEU	0.019	0.185	
EAS	Chr6:126422953..126469896	rs11963634..rs9375427	46944	16	YRI	0.019	0.093	CEU	0.037	0.167	
EAS	Chr8:82099819..82206515	rs7833919..rs9298347	106697	44	YRI	0.000	0.073	CEU	0.037	0.145	LOC392238
EAS	Chr8:139024425..139054833	rs6986279..rs6577869	30409	16	YRI	0.019	0.037	CEU	0.037	0.111	LOC401478
EAS	Chr10:91695196..91743274	rs10509597..rs2104483	48079	24	YRI	0.000	0.200	CEU	0.018	0.218	
EAS	Chr16:47969205..48226046	rs8058886..rs9938490	256842	80	YRI	0.000	0.074	CEU	0.019	0.204	ABCC11, LONP, SIAH1
EAS	Chr20:574657..583089	rs282152..rs282163	8433	6	YRI	0.000	0.224	CEU	0.000	0.138	TCF15
EAS	Chr22:41774895..41787863	rs138903..rs113515	12969	10	YRI	0.018	0.088	CEU	0.035	0.298	BZRP
CEU	Chr5:33991644..34012646	rs35406..rs35412	21003	17	YRI	0.034	0.119	EAS	0.000	0.220	MATP
CEU	Chr12:49117161..49164730	rs4424740..rs12581494	47570	14	YRI	0.036	0.236	EAS	0.036	0.200	LOC113251
CEU	Chr15:46081373..46155554	rs2470110..rs2433359	74182	42	YRI	0.000	0.019	EAS	0.000	0.130	NCKX5, MYEF2
CEU	Chr15:46160804..46237972	rs2459394..rs3784614	77169	32	YRI	0.000	0.017	EAS	0.000	0.186	MYEF2, LOC400369, SLC12A1
YRI	Chr4:153211294..153243151	rs1355413..rs17360461	31858	11	EAS	0.019	0.222	CEU	0.000	0.278	PET112L

The blocks with rMHH<0.05 and rHH<0.3 to both the reference populations are shown. Their positions and overlapping genes were referred to NCBI Build 34.

## Discussion

The previous LD-based methods using EHH are not applicable to already fixed alleles and also have reduced power for detecting alleles near fixation because relative EHH between different alleles is calculated as the statistic [22], [XPATH ERROR: unknown variable "next".]. However, to understand the genetic factors that determine the phenotypic differences between populations, we think it is better to focus on completely differentiated loci. For this purpose, we provide an alternative method of genome-wide scanning for swept loci that have reached fixation in a population. Here, the test population is compared with the reference population in HH and MHH in order to control for the heterogeneity of recombination rate across the human genome. In the procedure of this method, we partitioned genomic data into blocks depending on MHH in the test population. This enables us to detect those regions with low diversity and few past recombination events as large as possible. Such variable block size is adaptive also to detection of selection events under different situations, because the length of range showing LD decay depends on the strength of selection and on the frequency of the advantageous allele at the time that selective pressure began to act. By contrast, when a constant physical size of windows is used, summary statistics can greatly be confounded by the strength of selection, the initial frequency of the advantageous allele, and/or the heterogeneity of recombination rate [26], [44]. Although blocks were defined as having at least two SNPs with MHH ≥0.9 in this study, the definition can be changed. For example, when blocks are defined as having at least two SNPs with HH ≥0.5, we would expect to capture population-specific LD decay if the frequency of the advantageous allele had risen up to approximately 70% or more. Under this definition, indeed, we could detect strong signatures of selection not only on fixed loci but also on loci without fixation such as *LCT*, a representative selected gene in Europeans [22], [45] (data not shown).

In our method, rHH detects population-specific reduction in haplotype variation, which is a signature of a recent rapid increase in the frequency of an allele. Unlike relative EHH that examines each allele or core haplotype [24], rHH is applied to each block with a long range. Therefore, we can avoid independently testing adjacent loci in strong LD with each other. On the other hand, rMHH is an indicator of haplotype differentiation. Our simulation analyses suggested that rMHH has a higher ability to capture highly differentiated regions than maxF_ST_ does when the density of the typed SNPs is low and the selected polymorphism is not typed. However, maxF_ST_ would become effective if dense SNPs are available.

Summary statistics used for neutrality tests usually are confounded by population demographic history. Therefore, recent genome-wide scans for selection have resorted to outlier approaches based on empirical distribution, where a certain threshold is set (*i.e*., 1^st^ percentile of the empirical distribution) [13], [15], [18], [19]. Such approaches do not always guarantee high power to detect true selected loci [26]. In our method, the summary statistics, especially rMHH, are relatively robust against population demographic history when the test population has experienced fixation of a new advantageous mutation. Moreover, these statistics are relatively independent of the density of typed SNPs. Therefore, we can set the threshold for both statistics that assures approximately 90% detection power regardless of demographic history and SNP density. Using this approach, we can reduce false positives overall while maintaining high detection power for true selected loci. Given that the potential for false positives in a population can increase depending on that population's demographic history, the combination of the two statistics (and more than one reference population, if possible) allows one to efficiently narrow the candidates down to those with the strongest selection signatures. In this study, we observed only a fairly small number of candidates in the comparison between EAS and CEU, which indicates that the divergence time of the two populations is so short that new mutations could not reach fixation without strong selection. However, a number of candidates still remained when the non-African populations were tested against YRI. At the time of “out of Africa”, strong positive selections must have acted since the African and non-African populations show distinct phenotypic differences. To distinguish true selected loci from false positives, the length of block would also be a clue (Data S1).

Although recent advancement of statistical genetics and computer technology enables us to estimate haplotype phase in a large amount of genotype data [46], this procedure is still time consuming. In this study, however, even the approach using observed values of MHH and HH without phasing works fairly well. The conciseness and powerfulness, together with the applicability to data with relatively low SNP density, are convenient for analyses of genotype data produced by DNA microarray or other technologies. Thus, the present method can be a practical tool for future studies on other human populations and other species, such as domesticated animals and plants under artificial selection, in which evolutionary studies have only recently been performed [47], [48].

It should be noted again that many of candidate regions identified in this study might be false positives generated by genetic drift. Strong signatures can be dependent on a neighboring selected region. Nevertheless, the set of candidates here must include true selected loci that determine interpopulation phenotypic differences, since approximately 90% detection power was assured in each statistic used. Indeed, we observed the strongest signatures on genes previously reported to show association with traits such as pigmentation and earwax type. In addition, our method detected several candidates for selected genes that have previously been identified by scans using other methods and other data sets [19], [20]. Such genes are most likely to be true selected genes. To validate this, however, association studies between genotypes and phenotypes are required, using appropriate populations. It is also essential to carry out molecular analyses that identify location of selected polymorphisms, functions of genes in which selected polymorphisms are located, and effect of selected polymorphisms on gene functions. More importantly, this work may help reveal morphological and physiological characteristics of population specificity in detail. Such micro- and macro-level studies enable us to understand what factors have been selective pressures on modern humans and how we have adapted to them during the course of evolution.

## Materials and Methods

### Estimation of the detection power of rMHH and rHH with computer simulations

To estimate the detection power of rMHH and rHH, computer simulations were divided into two phases, divergent population phase and ancestral population phase (Fig. S1A). The divergent population phase was simulated with a forward-time simulation program for neutral or genic selection models, where the frequency of the advantageous allele was deterministically increased. In the ancestral population phase, a coalescent simulation program [29] for neutral models was used to create the initial state for the divergent population phase.

Since population size (N) and the number of SNPs (L) were restricted in the forward-time simulation due to the limitation of computational load, we set N = 1000 and L = 101 in the divergent population phase. In this simulation, we assumed no new mutation. The SNPs were placed at intervals of 2 kb and the center SNP (51^st^) was regarded as the selected polymorphism. Individual chromosomes of the founder generation at the population split (G = 0) were numbered, and the state of chromosomes at each generation was denoted by this number, not by allelic state. The regions simulated were 200 kb with 4Nr = 4×10^−4^, where r is recombination rate per generation per base. At 200 (0.2 N) and 300 (0.3 N) generations after split of the two populations, 120 chromosomes were sampled without replacement. The reference population with s = 0 (neutral) and the test population with s = 0 (neutral), 0.1 or 0.15 (selection; 2Ns = 200 or 300) were separately simulated, where s is selection coefficient. Different population size of the test population (Nt = 500) or the reference population (Nr = 500) was also examined to gauge the effect of demographic history on the statistics. We tested various initial frequencies of the advantageous allele (1, 20, 100, 200 and 400 per 2000 chromosomes). The simulation runs were replicated 100 times each for the test and reference populations. Since simulations were independent between the two populations, we used all the pairs of their combinations (100×100) for the analyses (Fig. S1A).

In the coalescent simulation for the ancestral phase, N was set to be 1000 and all the chromosomes (2N = 2000) were sampled. The region simulated was 500 kb with 4Nr = 4×10^−4^ and 4Nr = 6×10^−4^, where r is mutation rate per generation per base. We determined the selected polymorphism according to its derived allele frequency and, placing it at center, defined 100 surrounding windows of 2 kb. Then, the SNP with the highest minor allele frequency in each window was chosen as “genotyped SNPs” and relocated to be at constant intervals of 2 kb (Fig. S1B). The simulation runs were replicated 100 times. The result from each run in the ancestral population phase was regarded as an initial allelic state in the divergent population phase.

Finally, we obtained 120 chromosomes with allelic state and constructed 60 diploid individuals for each population. Blocks (containing at least two SNPs with MHH ≥0.9) were defined as described in the Results section. In the selection models, the selected SNP was not used for the analyses and only the block including or adjacent to the selected SNP was considered. In some cases, there was no such block, but those cases were also included in the power calculation as “undetected”. By contrast, every defined block in the simulated region was used in the neutral models.

### HapMap data

The individual genotype data from Phase I (release 16c.1) of the International HapMap Project were downloaded from the website (www.hapmap.org) [18]. Of the 270 individuals examined in the project, we used 180 unrelated individuals (60 from YRI; 60 from CEU; 30 from CHB; 30 from JPT). Autosomal SNPs typed in all the populations (883,697 SNPs) were analyzed in this study. We referred gene positions to NCBI Build 34.

### Neutral genome simulation

To obtain a set of genome-size data for 120 chromosomes each from the three human populations (YRI, CEU, and EAS) under neutral condition, a coalescent simulation was performed using the program and best-fitting demographic model of Schaffner *et al*. (Fig. S3A) [29]. In this simulation program, recombination rate can be varied within the region being simulated (500 kb). The simulation runs were replicated 5,400 times and all the regions simulated were connected to make chromosomes with the length of 2.7 Gbp.

To compare the HapMap data with the simulation data, correction of the ascertainment bias is essential. However, the accurate process of determining the ascertainment for the HapMap data is complicated. Consequently, since the SNP discovery rate depends on the minor allele frequency, we determined the probabilities that SNPs were “genotyped” according to the minor allele frequency spectra in the simulation and the HapMap (Fig. S3B). We obtained a set of “genotyped” SNP data from the simulation and constructed 180 individual diploid data from the haploid data.

## Supporting Information

Figure S1 Schema of simulation procedure for power estimation. (A) The simulation procedure was divided into two phases: divergent and ancestral population phases were performed with forward-time and coalescent simulation, respectively. (B) From the results of the coalescent simulation, a SNP was chosen as the selected SNP (orange circle) according to its derived allele frequency and the SNP with the highest minor allele frequency in each surrounding window (green circle) was also chosen and relocated to create the initial state for the forward-time simulation.
(0.27 MB TIF)

Figure S2 Initial frequency of the advantageous alleles and block size in the simulation. The case in which positive selection acts on a standing allele was simulated. Blocks were defined as regions with MHH≥0.9. Error bars denote the standard deviation.
(0.06 MB TIF)

Figure S3 Neutral genome simulation. (A) Demographic and genetic parameters in the simulation. T: time; N: effective population size; F: inbreeding coefficient; M: migration rate. (B) Correction of the ascertainment bias. The ratio of the number of SNPs in the HapMap to that in the simulation in each frequency is considered as the probability that SNPs were “genotyped.”
(0.44 MB TIF)

Figure S4 Scatter plots between rHH and physical length of blocks. (A) EAS (test) vs. YRI (reference). (B) EAS vs. CEU.
(0.53 MB TIF)

Data S1 Candidate regions of selective sweeps causing fixation
(0.37 MB XLS)
