# Supplementary figures and images for: A Practical Genome Scan for Population-Specific Strong Selective Sweeps That Have Reached Fixation
Source: PLoS One. 2007 Mar 14;2(3):e286. doi: 10.1371/journal.pone.0000286 (PMC1805687; doi:10.1371/journal.pone.0000286)

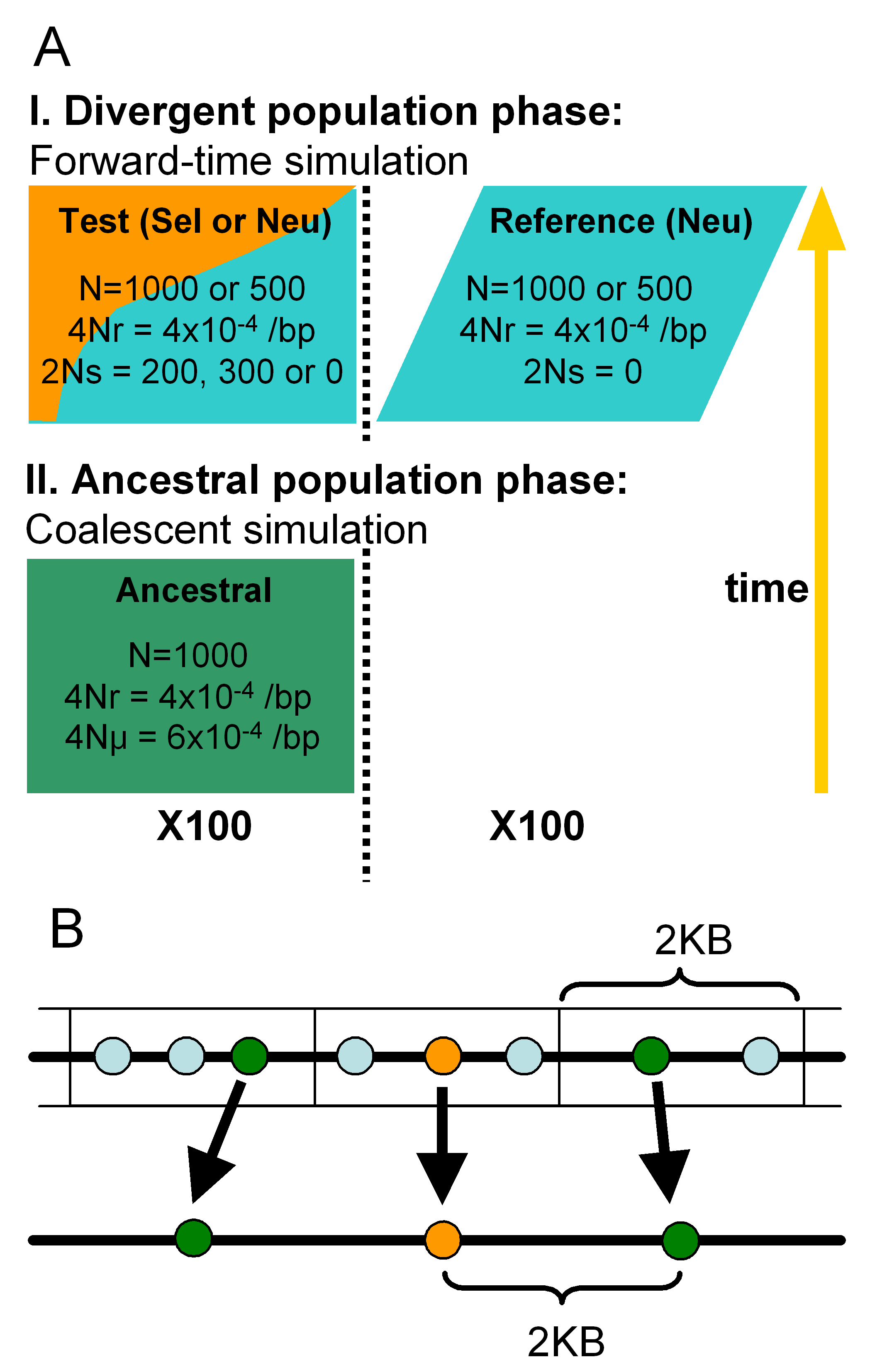

Supplement: Figure S1 — Schema of simulation procedure for power estimation. (A) The simulation procedure was divided into two phases: divergent and ancestral population phases were performed with forward-time and coalescent simulation, respectively. (B) From the results of the coalescent simulation, a SNP was chosen as the selected SNP (orange circle) according to its derived allele frequency and the SNP with the highest minor allele frequency in each surrounding window (green circle) was also chosen and relocated to create the initial state for the forward-time simulation. (0.27 MB TIF) [file pone.0000286.s001.tif]

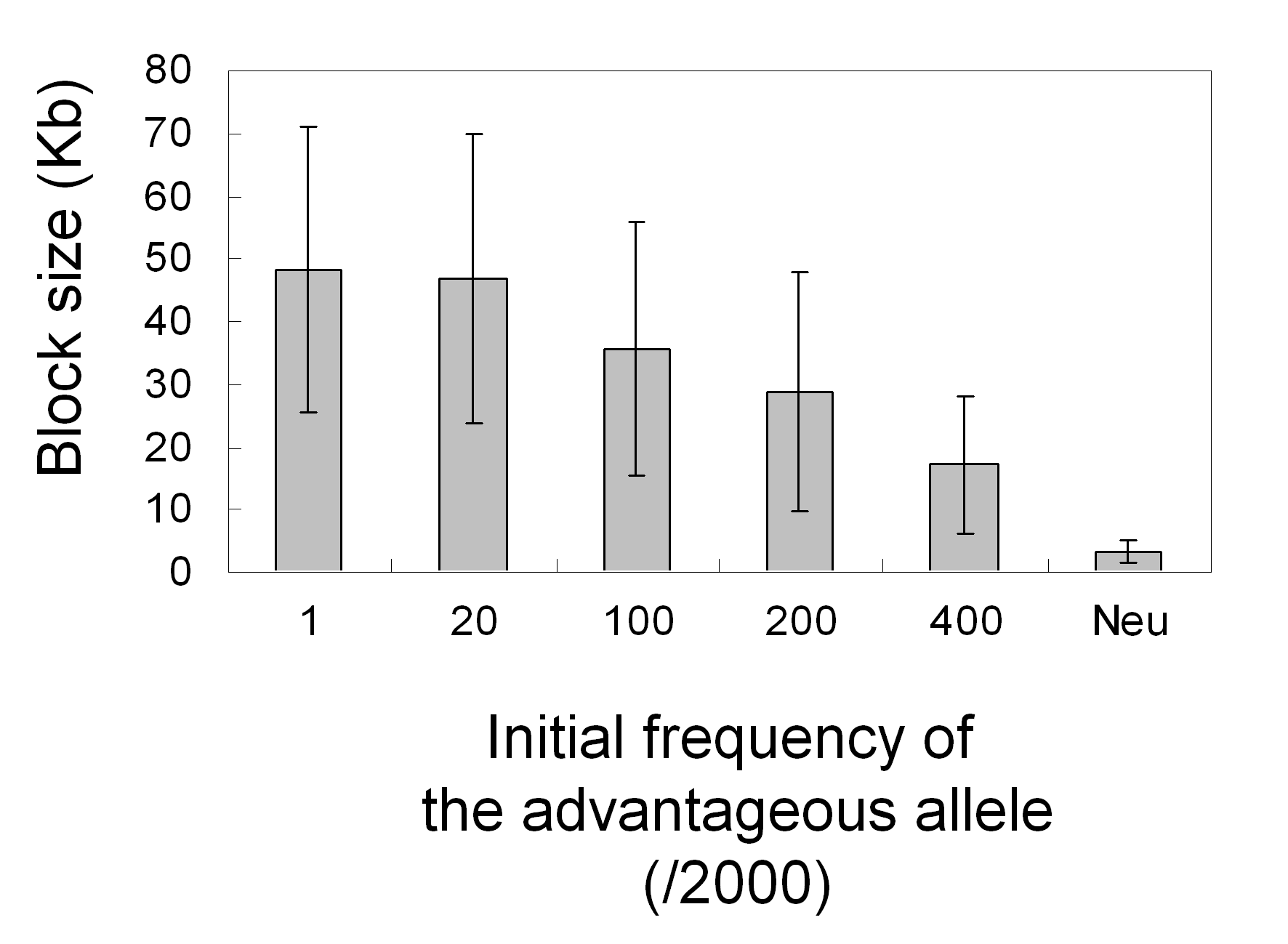

Supplement: Figure S2 — Initial frequency of the advantageous alleles and block size in the simulation. The case in which positive selection acts on a standing allele was simulated. Blocks were defined as regions with MHH≥0.9. Error bars denote the standard deviation. (0.06 MB TIF) [file pone.0000286.s002.tif]

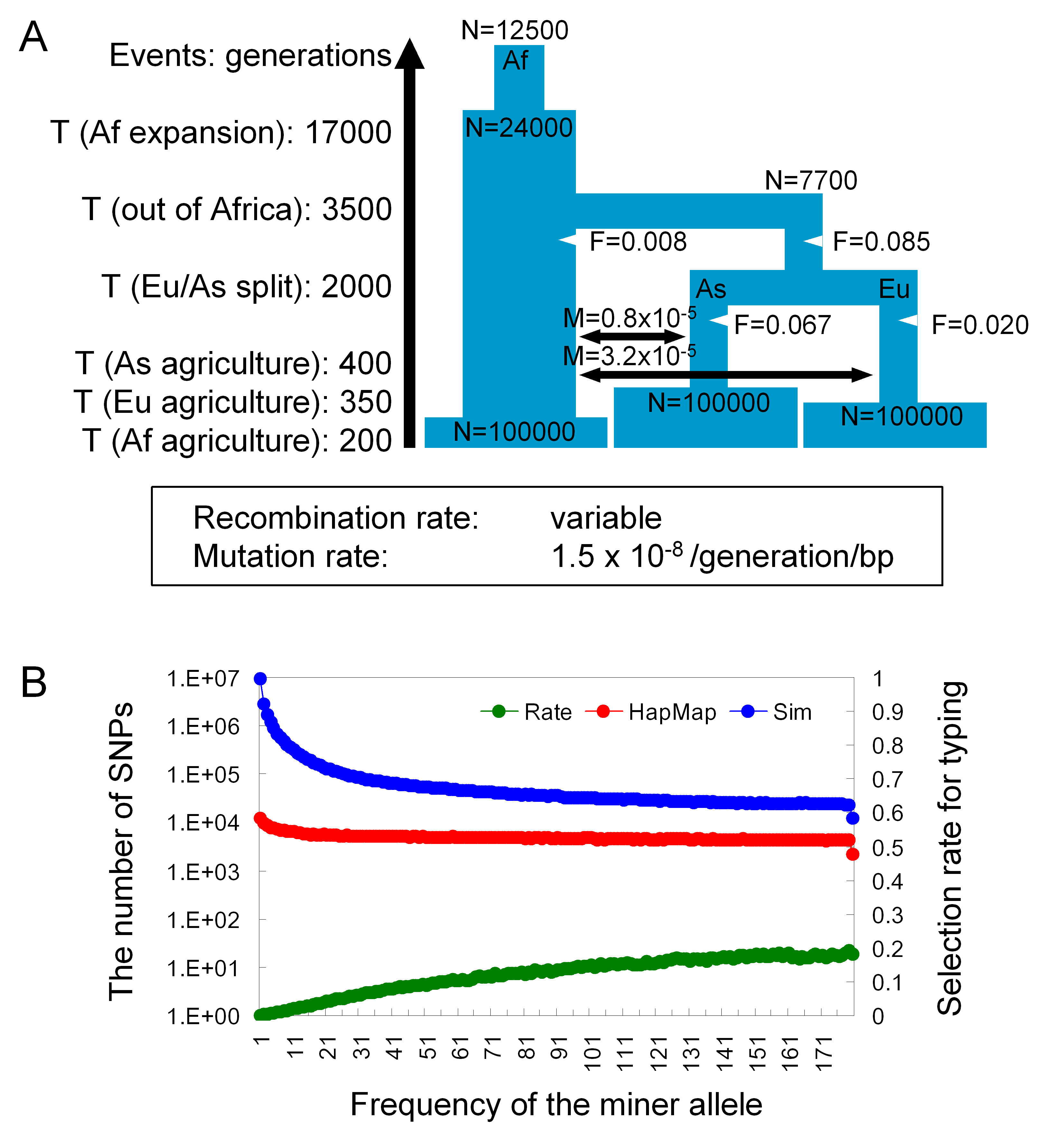

Supplement: Figure S3 — Neutral genome simulation. (A) Demographic and genetic parameters in the simulation. T: time; N: effective population size; F: inbreeding coefficient; M: migration rate. (B) Correction of the ascertainment bias. The ratio of the number of SNPs in the HapMap to that in the simulation in each frequency is considered as the probability that SNPs were “genotyped.” (0.44 MB TIF) [file pone.0000286.s003.tif]

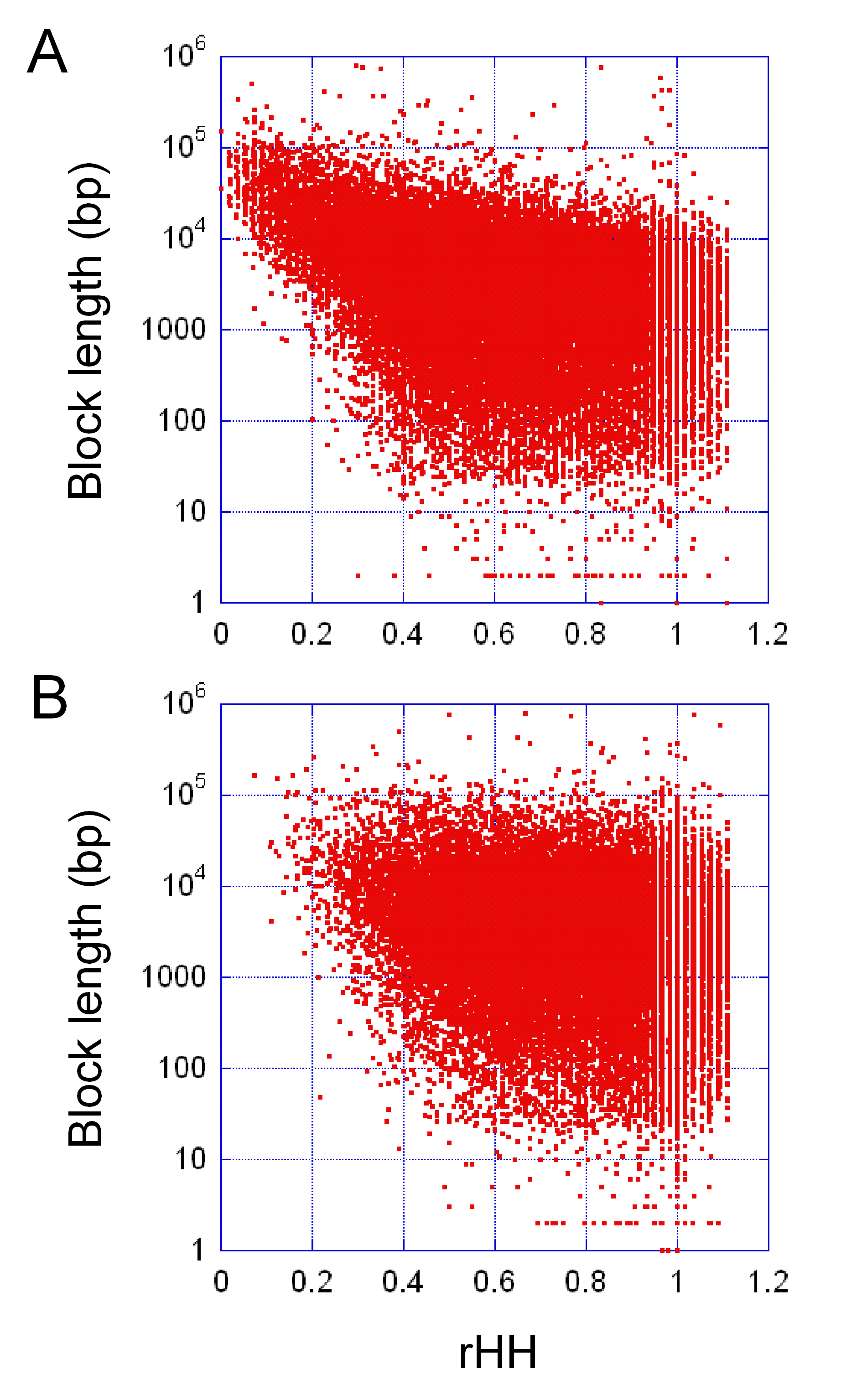

Supplement: Figure S4 — Scatter plots between rHH and physical length of blocks. (A) EAS (test) vs. YRI (reference). (B) EAS vs. CEU. (0.53 MB TIF) [file pone.0000286.s004.tif]
